# A New Method to Jointly Estimate the Mortality Risk of Long-Term Exposure to Fine Particulate Matter and its Components

**DOI:** 10.1038/srep18916

**Published:** 2016-01-06

**Authors:** Dan L. Crouse, Sajeev Philip, Aaron van Donkelaar, Randall V. Martin, Barry Jessiman, Paul A. Peters, Scott Weichenthal, Jeffrey R. Brook, Bryan Hubbell, Richard T. Burnett

**Affiliations:** 1 Environmental Health Science and Research Bureau, Health Canada, Ottawa, Ontario, Canada; 2 Department of Physics and Atmospheric Science, Dalhousie University, Halifax, Nova Scotia, Canada; 3 Harvard-Smithsonian Center for Astrophysics, Cambridge, Massachusetts; 4 Air Quality Assessment Section, Safe Environments Directorate, Health Canada, Ottawa, Ontario, Canada; 5 Department of Sociology, University of New Brunswick, Fredericton, New Brunswick, Canada; 6 Air Health Science Division, Safe Environments Directorate, Health Canada, Ottawa, Ontario, Canada; 7 Air Quality Research Division, Environment Canada, Downsview, Canada; 8 Dalla Lana School of Public Health, University of Toronto, Toronto, Ontario, Canada; 9 US Environmental Protection Agency, Office of Air Quality Planning and Standards, Research Triangle Park, North Carolina

## Abstract

Most studies on the association between exposure to fine particulate matter (PM_2.5_) and mortality have considered only total concentration of PM_2.5_ or individual components of PM_2.5_, and not the combined effects of concentration and particulate composition. We sought to develop a method to estimate the risk of death from long-term exposure to PM_2.5_ and the distribution of its components, namely: sulphate, nitrate, ammonium, organic mass, black carbon, and mineral dust. We decomposed PM_2.5_ exposure into the sum of total concentration and the proportion of each component. We estimated the risk of death due to exposure using a cohort of ~2.4 million Canadians who were followed for vital status over 16 years. Modelling the concentration of PM_2.5_ with the distribution of the proportions of components together was a superior predictor for mortality than either total PM_2.5_ concentration alone, or all component concentrations modelled together. Our new approach has the advantage of characterizing the toxicity of the atmosphere in its entirety. This is required to fully understand the health benefits associated with strategies to improve air quality that may result in complex changes not only in PM_2.5_ concentration, but also in the distribution of particle components.

Ambient fine particulate matter (PM_2.5_) is composed of a complex mixture of solids and liquids (smaller than 2.5 microns in aerodynamic diameter) derived from diverse sources (e.g., human activities, including fossil fuel combustion and industrial activities; and natural sources, including volcanic ash and pollens) that varies in space and time due to atmospheric chemistry, weather, and interactions between it and other pollutants in the atmosphere^1^. The composition of particulate matter, therefore, varies between and within regions of the world, countries, and urban and rural areas, and is influenced by such factors as climate, proximity to an ocean, agricultural activities, transportation activities, and kinds and quantities of point source emitters^2^. The proximity to sources also affects the nature of the mixture of constituents composing PM_2.5_ (mixing state) with a greater propensity for external mixtures of particle types closer to sources, and more homogeneity among particle types as an air mass ages and the particles undergo a greater degree of atmospheric processing. The major components of PM_2.5_ typically consist of: sulphate; nitrate; ammonium; chloride/sea salt; carbon – described variously as elemental carbon, organic carbon, and black carbon; crustal material, including dust and minerals; and, biological materials and organic mass^2^. The level of toxicity associated with PM_2.5_ is strongly affected by its mass and number concentrations, in addition to particle size, shape, chemical composition, and mixing state.

The vast majority of epidemiological studies on the association between long-term exposure to PM_2.5_ and mortality have considered only total concentrations of PM_2.5_, and not proportions or quantities of its individual components and constituents (e.g.,^3^,^4^,^5^,^6^,^7^,^8^,^9^). In this context, it is important to note that while previous studies (including those cohort studies noted above) have found consistently positive associations between long-term exposures to PM_2.5_ concentrations and all-cause and cause-specific mortality, the size of effect varied somewhat between places and by cause of death. For example, in their English cohort, Carey *et al.*^4^ found stronger associations with respiratory mortality than with cardiovascular mortality, which is inverse to findings in most US-based studies. Additionally, Carey *et al.*^4^ and Cesaroni *et al.*^5^ (in their Italian cohort) both reported notably weaker associations between long-term exposure to PM_2.5_ and all-cause mortality than those reported in Canadian^6^- and US-based studies^7^,^10^. In addition to differences in methodological choices between these studies (e.g., exposure assessment, covariate adjustment, period of follow-up) and population susceptibility to the effects of exposure, the differences in the findings may be due in part to variations in PM composition.

Given that most previous studies on the long-term effect of exposure have focused primarily on associations between mortality and overall PM_2.5_ concentrations, the kinds and sources of particles most responsible for long-term effects on health are not well known. Moreover, as outlined by Kelly and Fussell^1^: “having now firmly established associations between ambient PM and adverse health effects, the biggest gap in our knowledge of PM toxicity relates to which component(s) of ambient PM, and/or which of their physical and chemical characteristic(s), are responsible (p. 505).” There has, however, been recent interest in examining how the distribution of components modifies the effects of total PM_2.5_ concentration on mortality risk^11^.

The objective of this study is to develop a mathematical characterization of the combined association between fine particulate matter concentration and the distribution of its components that is suitable for use in survival analysis, which is the method used to estimate risk associated with exposure to long-term concentrations of ambient air pollution and mortality. We illustrate our modelling approach using recently developed estimates of PM_2.5_ concentration and the distribution of sulphate, nitrate, ammonium, organic mass, black carbon, and mineral dust for the world^12^. We assigned these estimates to a nationally-representative cohort of ~2.4 million Canadians who completed the 1991 census long form and were linked to mortality records to 2006^6^,^13^,^14^.

## Results

Our study included approximately 2.4 million subjects at baseline, nearly 300,000 of whom died of non-accidental causes during the 16 years of follow-up (Table 1). Nearly 60% of subjects lived in the East Central airshed (which encompasses all of southern Ontario and Quebec, including several of Canada’s largest cities). Only 4.9% of subjects self-identified as visible minorities, and 17.1% were immigrants.

**Figure 1:**
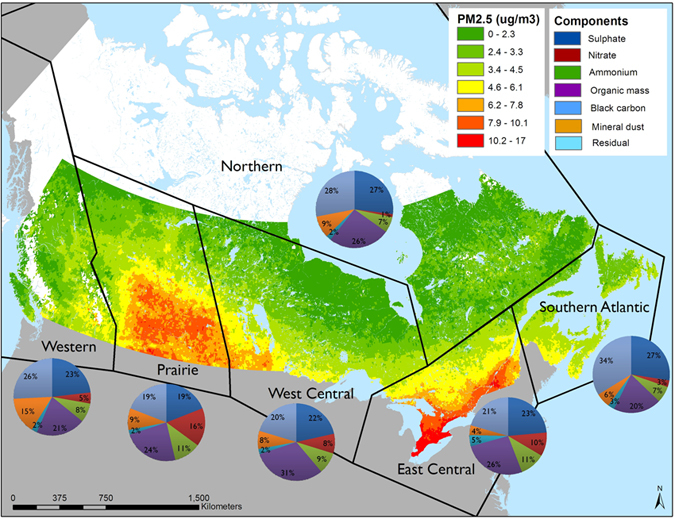
Median concentrations of PM_2.5_ (2001–2010) and relative component proportions by airshed. Map created in ArcGIS Desktop 10.2. ESRI, Redlands, CA.

At baseline, we found mean exposures to PM_2.5_ of 8.3 μg/m^3^. As depicted in Fig. 1, the highest concentrations were located in south-western Ontario, especially in the Windsor-to-Quebec City corridor, and in the western prairies. We present in Supplemental Material, Fig. 1 a map of the individual component concentrations across Canada. Among the six components, organic mass and sulphate contributed the largest proportions of PM_2.5_ (e.g., mean proportions at baseline of 0.21 and 0.23, respectively), while mineral dust and black carbon represented the smallest contributions (e.g., mean proportions at baseline of 0.05 each) (see Table 2 for complete distributions).

The Western airshed was characterised by a relatively high proportion of mineral dust (15%) and a low proportion of nitrate (5%). The Prairie airshed was characterised by the highest proportion of nitrate (16%), balanced by the lowest proportions of both black carbon (19%) and sulphate (19%). The West Central airshed had the highest proportion of organic mass (31%), whereas the Southern Atlantic airshed had the lowest proportion of organic mass (20%) and the highest proportion of black carbon (34%). The Northern airshed was composed of only 1% nitrate. The component concentrations are all highly correlated with each other and with total PM_2.5_ concentration (r > 0.65) except for dust (r < 0.50) (Table 3).

We present results for total PM_2.5_ concentration and each component concentration individually for non-accidental and cardio-metabolic deaths in Table 4. For each model, the logarithm of total PM_2.5_ concentration and the logarithm of component concentration was examined. In addition, we present the Akaike Information Criterion (AIC) as a measure of model fit adjusted for the number of variables in the model. Here, lower AIC values represent improved prediction of mortality.

First, the associations between mortality and total PM_2.5_ concentration, and between mortality and each component concentration individually, were positive and statistically significant based on coefficient to standard error ratios greater than two for non-accidental (left-hand models; top half of Table 4) and cardio-metabolic (left-hand models; bottom half of Table 4) mortality; the lone exception being dust. This result suggests that not all of the total PM_2.5_ concentration association with mortality can be explained by any single component. Total PM_2.5_ concentration, however, was a better predictor of non-accidental mortality compared to any component concentration individually, as measured by the AIC (Table 4).

Next, models consisting of all six components examined simultaneously (middle models of Table 4) were better predictors of mortality (both causes of death) than was total PM_2.5_ alone. This finding suggests that understanding the complete composition of the atmosphere improves mortality prediction. The coefficients in the multi-component concentration models were highly unstable compared to those in the single component concentration models, due likely to the very high correlations between component concentrations (Table 3). The high correlations are due, in part, to the manner in which these component concentrations were calculated as the product of total concentration times the component proportion. Thus, in areas of high or low overall PM_2.5_ concentration, the component concentration has a tendency to also be relatively high or low.

Third, we developed a methodology that somewhat elevates this confounding issue by decomposing total PM_2.5_ concentration into its component proportions. Here, the joint total concentration plus the distribution of component proportions model (right-hand models, Table 4) was a better predictor than was PM_2.5_ concentration alone, each of the component concentrations separately, and all six together jointly for both non-accidental (AIC = 5,731,809.8) and cardio-metabolic (AIC = 2,207,515.9) mortality. Additionally, we calculated likelihood ratio tests comparing models with PM_2.5_ alone and those with PM_2.5_ plus component proportions for models with both causes of death. Here we found for deaths from non-accidental and cardio-metabolic causes chi-square values of 42.6 and 48.8, respectively, with six degrees of freedom that resulted in p-values < 0.0001 in both cases. The evidence from these AIC and likelihood ratio tests support modelling total PM_2.5_ concentration and the distribution of its components together.

**Figure 2:**
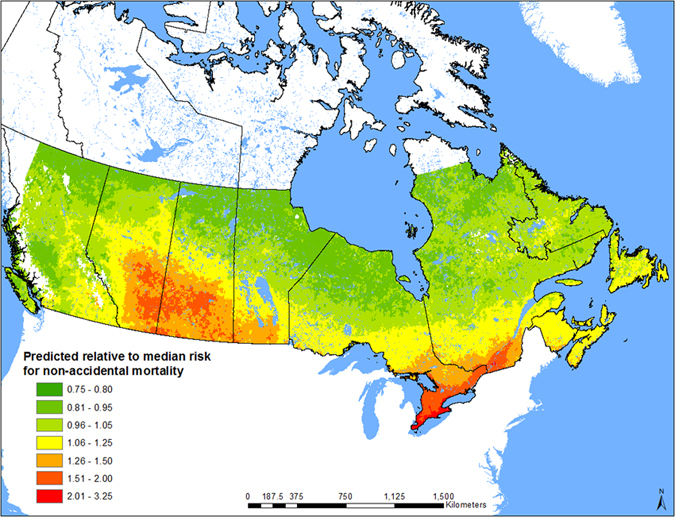
Predicted relative to median risk for non-accidental mortality. Map created in ArcGIS Desktop 10.2. ESRI, Redlands, CA.

We present in Fig. 2 the spatial distribution of predicted, relative to median risk estimates, for non-accidental mortality in each 10 × 10 km grid cell across Canada. As would be expected given the results of the models described earlier, these spatial patterns of risk largely mirror the patterns of PM_2.5_ concentrations as presented in Fig. 1. One notable difference between the patterns depicted in these two figures is the relatively moderate estimates of risk throughout the maritime provinces of eastern Canada, compared to the relatively low estimates of PM_2.5_ in these areas. The somewhat elevated levels of risk in these provinces may be due to the relatively high proportions of sulphate and black carbon of the PM_2.5_ in that region (as noted in the pie charts of Fig. 1).

We note that the influence of any single component proportion cannot be evaluated in isolation of the other components. Each component is only assessed appropriately by considering the joint change in concentration and the component distribution together, as can be done using chemical transport models. Here, specific changes in pollutant sources, for example, can be modelled simultaneously.

**Figure 3:**
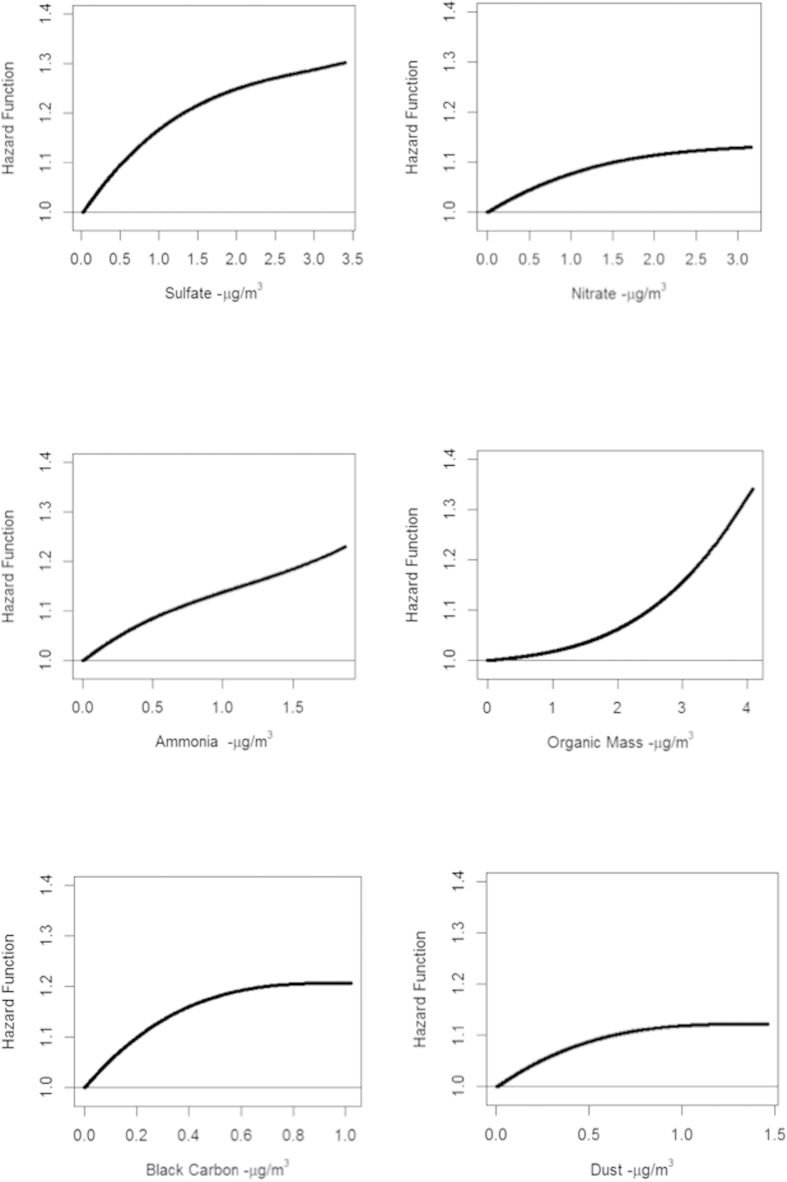
Smoothed plots of predicted risk of non-accidental mortality and component concentrations.

We present in Fig. 3 the smoothing spline representation of our predicted hazard function for non-accidental mortality vs. each individual component concentration. Here we can see that the risk of mortality is positively associated with each component concentration, possibly due to the role total PM_2.5_ concentration plays in both our hazard function and component concentrations. We also observed, however, that the range in predictions of the smooth representation of our hazard function is greatest for sulphate and organic mass, moderate for ammonia and black carbon, and least for nitrate and dust. These observations suggest an ordering of the influence of the individual components on our predicated hazard function.

## Discussion

We found that an estimate of total PM_2.5_ concentration was a better predictor of mortality than was an estimate of exposure to any individual component of PM_2.5_. Moreover, we found our best predictions of mortality for models with both the total concentration of PM_2.5_, along with the relative proportions of the six components, together. Knowledge of the relative proportions of the components that make up the total concentrations of particulate matter improved our ability to predict mortality.

A host of studies conducted around the world have now demonstrated that long-term exposures to PM_2.5_ are associated with increased mortality^3^,^4^,^5^,^6^,^7^,^9^,^10^,^15^,^16^. Moreover, several studies have attempted to identify the constituents or components of PM_2.5_ that may be most responsible for PM_2.5_-related health outcomes^17^,^18^,^19^,^20^,^21^,^22^,^23^.

In this paper, we took a different approach in that we examined how the association between PM_2.5_ and mortality is modified by the distribution of components at a given geographic location. In particular, we developed a new mathematical model to decompose PM_2.5_ concentrations into total concentration and the distribution of components, suitable for use in standard survival models.

Initially we examined the association between the concentration of each component and mortality, where component concentration was constructed as the product of total PM_2.5_ and component proportions. Because each component concentration is a function of total PM_2.5_ concentration, the component concentrations were highly correlated, thus making it difficult to estimate mortality associations for each component simultaneously.

To address this issue, we decomposed PM_2.5_ into total concentration plus the distribution of component proportions and jointly estimated mortality associations for these new variables. We also demonstrated that such decomposition improved our ability to predict mortality compared to PM_2.5_ concentration alone or by simultaneously modelling all component concentrations together. This is an important advance as the impetus behind the assessment of PM_2.5_ components is driven largely by the assumption that knowledge of individual constituents may allow for more efficient regulation if the most important components of PM_2.5_ can be targeted for reduction.

A key strength of our study is the large size of our nationally-representative cohort. CanCHEC is one of the largest cohort studies in the world, and includes data on many potential risk factors for mortality. Our exposure models covered all but the largely unpopulated northern regions of the country. As such, we were able to include subjects from every province and from urban and rural areas across Canada.

A noted limitation of our cohort dataset is that personal covariates were available only at baseline. Subjects were followed for vital status, but not for socio-demographic information (e.g., income, employment status). A related limitation of our analysis is that our exposure models described a fixed period of time (i.e., mean concentrations of PM_2.5_ over the period 2001–2010), and therefore may not accurately describe longer-term patterns of pollutant concentrations. It is also important to note that other components of PM_2.5_, such as metals, were not examined in this study and may play an important role in overall PM_2.5_ toxicity owing to their ability to cause oxidative stress^24^,^25^. Uncertainty in the PM_2.5_ components may also impact the resultant model, particularly where one component is better represented than another. Estimates of sulphate, for example, show particularly good agreement with ground observations (r = 0.95) suggesting associations with this component will be better captured than other components. If possible, future studies should expand this kind of analysis to include other potentially important particle constituents as such data become available.

More generally, the method described in this study can be used to estimate the population health burden of disease associated with specific strategies to improve air quality. Such strategies may alter the components in complex ways. We suggest risk models consisting of multiple components estimated simultaneously along with total concentration be used over models with either concentration alone, or component concentrations individually or even simultaneously. This approach provides policy analysts with a powerful tool given that estimates of changes in the atmospheric mix of pollution can be made throughout the world. The associations between mortality and PM_2.5_ and its composition, however, may differ in other parts of the world where populations have differences in susceptibility to the effects of exposure, and where overall concentrations of PM_2.5_ are much higher (as effects may not be linear). We recommend that similar analyses be conducted with cohort studies of ambient air pollution throughout the world in order to obtain more stable and generalizable estimates of mortality risk associated with exposure to the atmospheric mixtures of particle phase pollution.

## Methods

### The study cohort

The Canadian Census Health and Environment Cohort (CanCHEC) has been described in detail previously^6^,^13^,^14^. CanCHEC is a population-based cohort of subjects who were 25 years of age and older at baseline; a usual resident of Canada on the Census reference day (June 4, 1991); not a resident of an institution such as a prison, hospital, or nursing home; and among the 20% of Canadian households selected for enumeration with the long-form Census questionnaire. Subjects in CanCHEC were linked to the Canadian Mortality Database using deterministic and probabilistic linkage methods from June 4, 1991 through December 31, 2006^13^. The date of death and the underlying cause of death were extracted from death certificates coded to the International Classification of Diseases, 9th Revision (ICD-9)^26^ for deaths before 2000, and to ICD-10^27^ for those that occurred from 2000 onward. We also linked annual place of residence (six-character postal code) using Historical Tax Summary Files from 1990 through 2006^13^. In urban areas, six-character residential postal codes correspond to one side of a city block or to a single apartment building; in rural areas they may represent a larger area. We excluded recent immigrants (those immigrating in the eight years prior to baseline) given that they tend to have notably different health behaviours and outcomes^28^ and may have notably different past exposures, than the native-born Canadian population.

### Assignment of exposure to ambient air pollution

We assigned estimates of concentrations of PM_2.5_ and the relative proportions of several constituent components to the representative point of each subject’s residential postal code for every available year between 1990 and 2006 (i.e., beginning one year prior to baseline). A subject’s exposure was coded as missing in years for which no residential postal code was available, which could indicate that the subject had left Canada, moved to an institution, or that they had not filed an income tax return that year.

We derived estimates of PM_2.5_ from observations from three satellite instruments (MISR, MODIS, and SeaWiFS) to represent annual concentrations during the period 2001–2010 on a grid with a spatial resolution of approximately 10 km × 10 km and included coverage up to 60°N (i.e., excluding the northern regions of Canada)^29^. These PM_2.5_ estimates were generated by relating satellite retrievals of aerosol optical depth (AOD) to near-surface PM_2.5_ using the AOD to PM_2.5_ relationship calculated from the GEOS-Chem chemical transport model Significant agreement was found with North American ground-based monitors (r = 0.76). Inter-annual variation in PM_2.5_ between 2001 and 2010 was inferred from two radiometrically stable satellite instruments (MISR and SeaWiFS) following the same methodology^30^.

We estimated the proportion of PM_2.5_ attributed to six components, namely: sulphate, nitrate, ammonium, organic mass, black carbon, and mineral dust. The proportion attributed to each component was calculated with the GEOS-Chem chemical transport model as described in Philip *et al.*^12^ by linearly interpolating the native resolution of 50 km by 66.7 km grid resolution to the 10 km by 10 km of the gridded satellite observations and applying the distribution of components to the estimated PM_2.5_ concentration. We applied a species-dependent sampling correction and an additional correction/capping to avoid unrealistic retrievals. Evaluation with ground-based observations across the United States and Canada indicated significant agreement for all components. The remaining percentage was assigned to a residual component such that percentages summed to unity.

For each subject and for each year of follow-up we assigned a single-year lagged estimate of exposure to PM_2.5_ and the proportion of each component. This method of exposure assignment allowed us to incorporate into our models variability in exposure associated with annual residential mobility patterns. The spatial structures of the exposure surfaces were not assessed in a time-dependent manner and thus variation in exposure for each subject was attributed solely to residential mobility.

### Decomposition into distribution of particulate components

We propose to estimate the joint association between mortality and long-term exposure to concentrations of fine particulate matter, accompanied by the distribution of components. That is, to consider simultaneously the total concentration of PM_2.5_ and its makeup. In an earlier study with this cohort, we observed the logarithm of PM_2.5_ to be a stronger predictor of mortality than PM_2.5_ itself 6. We therefore develop our approach to modelling PM_2.5_ and the component proportions based on the logarithm of PM_2.5_ concentration.

To do this, we set up the following notation. Let 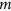
denote the PM_2.5_ concentration in μg/m^3^ and let 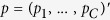 be a vector of proportions of total concentration attributable to the 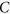
components of interest with 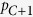 denoting the proportion of the residual component. Total PM_2.5_ concentration can then be decomposed into the concentration times a product of the component proportions normalized to unity by its geometric mean in the following manner:


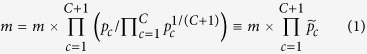


with 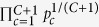 the geometric mean of the proportions, since 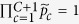 by construction. Here,


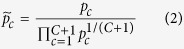


is the ratio of the 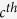 component proportion to its geometric mean. We then have


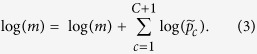


The corresponding part of the hazard function that involves fine particulate matter and its components in the Cox proportional hazards model is


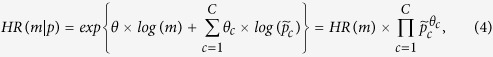


with 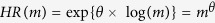 the hazard ratio model for total concentration in a model with the component proportions. Here 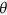 is an unknown risk parameter associated with total concentration and the 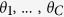 are the corresponding unknown parameters for the *C* components, which relate the deviation in risk for each component to the risk of total concentration on the logarithmic scale. This approach allows us to modulate the hazard ratio based on spatial variations in component proportions.

Note that the residual component is not included in the survival model due to over specification, since, given 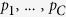, 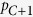 is known. Estimates of the unknown regression parameters are obtained by fitting a Cox proportional hazards regression model with all the mortality risk factors included in Table 1, PM_2.5_ concentration (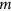), and the normalized component proportions (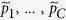).

The manner in which the components modulate the hazard ratio is complex, with an increase in any single component proportion requiring decreases in the others, since all component proportions must sum to unity. Note that for component proportions greater than the geometric mean of all component proportions 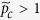, and if a proportion is less than the geometric mean of all component proportions then 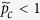. To interpret our model we need to consider four cases:

1. If 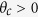 and 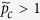, 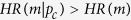 and will increase as 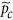 increases,
2. If 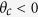 and 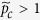, 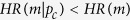 and will decrease as 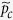 increases,
3. If 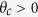 and 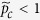, 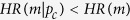 and will increase as 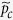 increase,.
4. If 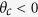 and 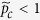, 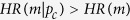 and will decrease as 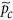 increases,

where 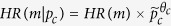 is the hazard ratio for concentration and a single marginal component within the joint model. (Note: We include as Supplemental Information the method and formulas for deriving the PM_2.5_ and distribution of components decomposed for linear hazard models).

### Main statistical analyses

We used Cox proportional hazards models to estimate the associations between exposure estimates and mortality. We estimated hazard ratios (HRs) stratified by sex, five-year age groups from age 25 to 89, and by regional airshed. We restricted our study to subjects less than 90 years of age due to potential inaccuracies in record-linkages among older subjects (e.g., the address reported on the annual income tax filings of older subjects may reflect those of next of kin, or of institutional facilities). A collaborative approach known as the Air Quality Management System (AQMS) has been developed recently by Canadian federal, provincial, and territorial governments for reducing air pollution in Canada. Among other tasks, the AQMS has delineated six regional airsheds that cut across jurisdictional boundaries in Canada and that have similar air quality characteristics or air movement patterns (Personal communication with Jeff Brook, Environment Canada, December 2014). We stratified by airshed with the intention of explaining broad spatial variation in both mortality and air pollution while still maintaining some variability in exposure within each airshed.

We adjusted our models for aboriginal ancestry, visible minority status, immigrant status, marital status, highest level of education, employment status, occupational classification, and quintiles of household income (see Table 1 for coding). We also calculated time-varying contextual variables adjusted for regional variations across Canada (i.e., census division (CD) means subtracted from census-tract (CT) means) describing the proportion of unemployed adults, the proportion of adults who had not completed high school, and the proportion of individuals in the lowest income quintile. Census tracts correspond roughly to the size of a neighbourhood and census divisions correspond roughly to the size of a city or county. These time-varying contextual covariates describe characteristics of subjects’ current residential location during each year of follow-up.

We developed models for two causes of mortality, namely all non-accidental causes (ICD-9 codes <800; ICD-10 codes starting with letters A through R) and cardio-metabolic diseases (i.e., circulatory plus diabetes; (ICD-9 codes: 390–459 and 250; ICD-10 codes: I00-I99 and E10-E14)). Subjects were censored at time of death or if they were lost to follow-up due to end of study period or lack of postal code information. All analyses were conducted in SAS Version 9.3.

We also estimated the association between the logarithm of each component concentration individually (i.e., log(PM_2.5_*component proportion)) and mortality for the purpose of presenting results in a manner used by previous investigators. In addition, we present results based on all six component concentrations together in the same survival model.

We calculated Pearson correlations (r) between the component concentrations and untransformed PM_2.5_ concentration as assigned to subjects at baseline stratified by airshed. Here, we calculated the correlations by airshed separately and then determined a weighted average of the airshed-specific correlations with weights proportional to the number of subjects in each airshed.

Lastly, we calculated the predicted risk estimate in each 10 × 10 km grid cell (n = 75,118 cells) across Canada based on the local concentration of PM_2.5_ and relative proportions of the components. We then estimated the relationship, using smoothing splines, between the predicted hazard function for non-accidental mortality and each component concentration in order to understand how our complex hazard model related to the more standard representation of component information, namely each component concentration.

### Approval

The Canadian Census Health and Environment Cohort (CanCHEC) received approval by the Statistics Canada Policy Committee (reference number 012-2001) after consultation with the Statistics Canada Confidentiality and Legislation Committee, Data Access and Control Services Division, and the Federal Privacy Commissioner. This approval is equivalent to that of standard research ethics boards.

## Additional Information

**How to cite this article**: Crouse, D. L. *et al.* A New Method to Jointly Estimate the Mortality Risk of Long-Term Exposure to Fine Particulate Matter and its Components. *Sci. Rep.* **6**, 18916; doi: 10.1038/srep18916 (2016).

## Supplementary Material

Supplementary Information

## Figures and Tables

**Table 1:**
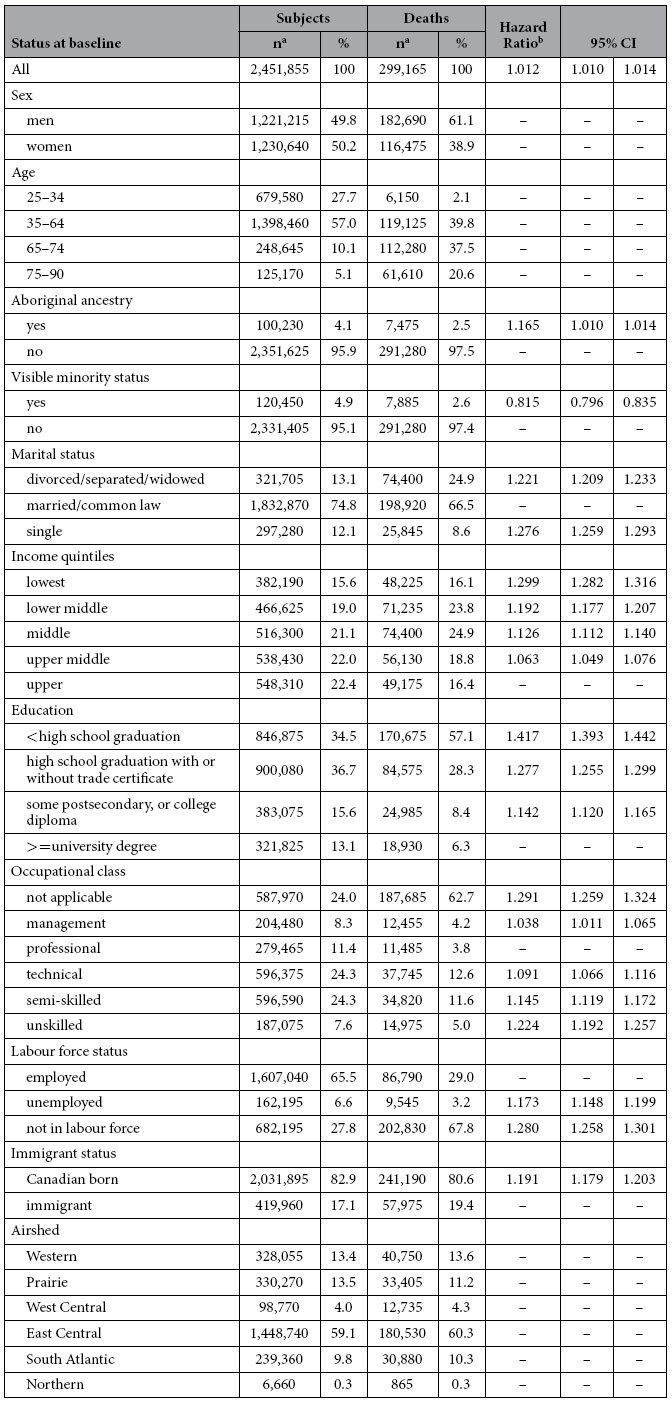
Descriptive statistics of cohort subjects at baseline and fully-adjusted hazard ratios for risk factors included in the survival models for all non-accidental causes of death.

^a^subject counts rounded to nearest 5; percentages based on original values.

^b^from models adjusted for the following personal covariates: PM_2.5_, aboriginal ancestry, visible minority status, highest level of education, employment status, occupational class, immigrant status, marital status, and income quintile; and the following contextual covariates: CD and CT-CD % of immigrants, % of adults without high school diploma, % of subjects in lowest income quintile; models stratified by age, sex, and airshed; hazard ratios per μg/m^3^ increment in exposure to PM_2.5_.

**Table 2:** Distributions of exposures at baseline.

		Components: proportions					
	PM_2.5_ (μg/m^3^)	Sulphate	Nitrate	Ammonium	Particulate organic mass	Black carbon	Mineral dust
Mean	8.26	0.21	0.12	0.10	0.23	0.05	0.05
Max	17.00	0.38	0.38	0.16	0.39	0.07	0.20
95%	13.90	0.29	0.29	0.14	0.29	0.07	0.10
90%	12.60	0.25	0.25	0.13	0.28	0.06	0.08
75%	10.80	0.22	0.22	0.11	0.27	0.06	0.07
Median	8.00	0.20	0.20	0.11	0.23	0.06	0.03
25%	5.60	0.19	0.19	0.10	0.19	0.03	0.03
10%	4.20	0.18	0.18	0.08	0.15	0.02	0.02
5%	3.60	0.18	0.18	0.07	0.13	0.02	0.02
Min	1.20	0.14	0.14	0.02	0.03	0.00	0.02

**Table 3:** Pearson correlations between concentrations (μg/m^3^) of PM_2.5_ and the six components, as assigned to subjects at baseline and stratified by airshed.

	PM_2.5_	Sulphate	Nitrate	Ammonium	Particulate organic mass	Black carbon	Mineral dust
PM_2.5_	1	0.93	0.89	0.96	0.81	0.91	0.42
Sulphate		1	0.75	0.90	0.72	0.80	0.47
Nitrate			1	0.93	0.66	0.89	0.23
Ammonium				1	0.76	0.90	0.40
Particulate organic mass					1	0.71	0.49
Black carbon						1	0.30
Mineral dust							1

**Table 4:** Results from single and-multi-component models for deaths from non-accidental causes (n = 299,165) and cardio-metabolic causes (n = 116,725).

Cause of Death	Pollutant	Single-component concentration models			Multi-component concentration models			PM_2.5_ + component distribution models		
		AIC	Estimate	Std. Error	AIC	Estimate	Std. Error	AIC	Estimate	Std. Error
Non-accidental		(5,731,800+)			(5,731,800+)			(5,731,800+)		
					25.4			9.8		
	PM_2.5_	40.5	0.084	0.009					0.080	0.012
	Sulphate	43.9	0.092	0.010		0.127	0.022		0.025	0.043
	Nitrate	84.7	0.030	0.004		0.004	0.012		−0.039	0.023
	Ammonium	76.0	0.049	0.006		0.021	0.028		0.033	0.027
	Organic mass	117.5	0.031	0.007		−0.032	0.015		−0.053	0.020
	Black carbon	83.9	0.037	0.005		−0.009	0.014		−0.049	0.025
	Dust	135.7	0.010	0.009		−0.042	0.012		−0.068	0.020
									
Cardio-metabolic		(2,207,500+)			(2,207,500+)			(2,207,500+)		
					22.6			15.9		
	PM_2.5_	52.7	0.133	0.014					0.143	0.02
	Sulphate	46.5	0.153	0.015		0.235	0.036		0.177	0.068
	Nitrate	87.9	0.051	0.007		0.034	0.019		0.025	0.037
	Ammonium	76.0	0.085	0.010		0.024	0.045		0.054	0.043
	Organic mass	112.8	0.065	0.011		0.014	0.024		0.017	0.032
	Black carbon	100.8	0.055	0.008		−0.091	0.023		−0.086	0.041
	Dust	143.9	0.023	0.014		−0.085	0.019		−0.077	0.032
