# Supplementary material for: A New Method to Jointly Estimate the Mortality Risk of Long-Term Exposure to Fine Particulate Matter and its Components
Source: Sci Rep. 2016 Jan 6;6:18916. doi: 10.1038/srep18916 (PMC4702114; doi:10.1038/srep18916)

A New Method to Jointly Estimate the Mortality Risk of Long-Term Exposure to Fine Particulate Matter and its Components

Dan L. Crouse, Sajeev Philip, Aaron van Donkelaar, Randall V. Martin, Barry Jessiman, Paul A. Peters, Scott Weichenthal, Jeffrey R. Brook, Bryan Hubbell, Richard T. Burnett

Supplemental Information

Here we derive the mass and distribution of components decomposed for linear hazard models.

Let m denote the total fine particulate mass in $\mu g / m^3$ and further let $p = (p_1,..., p_C)'$ be a vector of proportions of total mass attributable to the C components of interest and let p_{C+1} be the proportion of the residual component. Total mass can then be decomposed into the sum of mass and the difference between the components and mass as

$$m = m + m \times \sum_{i=1}^{C+1} \widetilde{p}_c ,$$

where $\widetilde{p}_c = p_c - \frac{1}{C+1}$ is the component proportion centred such that $\sum_{c=1}^{C+1} \widetilde{p}_c = 0$. The Cox proportional hazard model has the form

$$h^{(s)}(t|x, m, \widetilde{p}) = h_0^{(s)}(t) \exp\left\{\beta' x + \gamma \times m + \sum_{c=1}^{C} \gamma_c \times m \times \widetilde{p}_c \right\} \equiv h_0(t) \exp\{\beta' x\} \times HR(m|\widetilde{p}),$$

where $h_o^{(s)}(t)$ is the baseline hazard function of followup time t and strata s, x is a vector of known mortality risk factors such as income, occupation, and education with corresponding unknown parameter vector β. Strata are defined by five year age groups, sex, and airshed in our cohort. Here γ is an unknown risk parameter associated with total mass and the $\gamma_1,..., \gamma_C$ are the corresponding unknown parameters for the C components which relate the deviation in risk for each component to the risk of total mass. Note that the residual component is not included in the survival model due to over specification since given $p_1,..., p_C$, p_{C+1} is known. Estimates of the unknown regression parameters are obtained by fitting a Cox proportional hazards regression model with all the mortality risk factors included in x, particulate mass, m, and the component masses $m \times \widetilde{p}_1,..., m \times \widetilde{p}_C$.

The hazard ratio function of fine particulate matter and its components is denoted by

$$HR(m|p) = \exp\left\{m \times \left(\gamma + \sum_{c=1}^{C} \gamma_c \times p_c \right)\right\} = \exp\left\{m \times \gamma \times \left(1 + \sum_{c=1}^{C} \frac{\gamma_c}{\gamma} \times p_c \right)\right\},$$

$$= HR(m)^{\left(1 + \sum_{c=1}^{C} \frac{\gamma_c}{\gamma} \times p_c \right)}$$

where $HR(m) = \exp(\gamma \times m)$ represents the hazard ratio for total mass. We thus interpret the hazard ratio as modulating the mass hazard ratio by a power that is a function of the proportion of mass attributable to each component, p_c, and the ratio of the component risk parameter to the mass risk parameter $\frac{\gamma_c}{\gamma}$.

Supplemental Material, Figure 1: Concentrations of $PM_{2.5}$ components ($\mu g/m^3$). Map created in ArcGIS Desktop 10.2. ESRI, Redlands, CA.

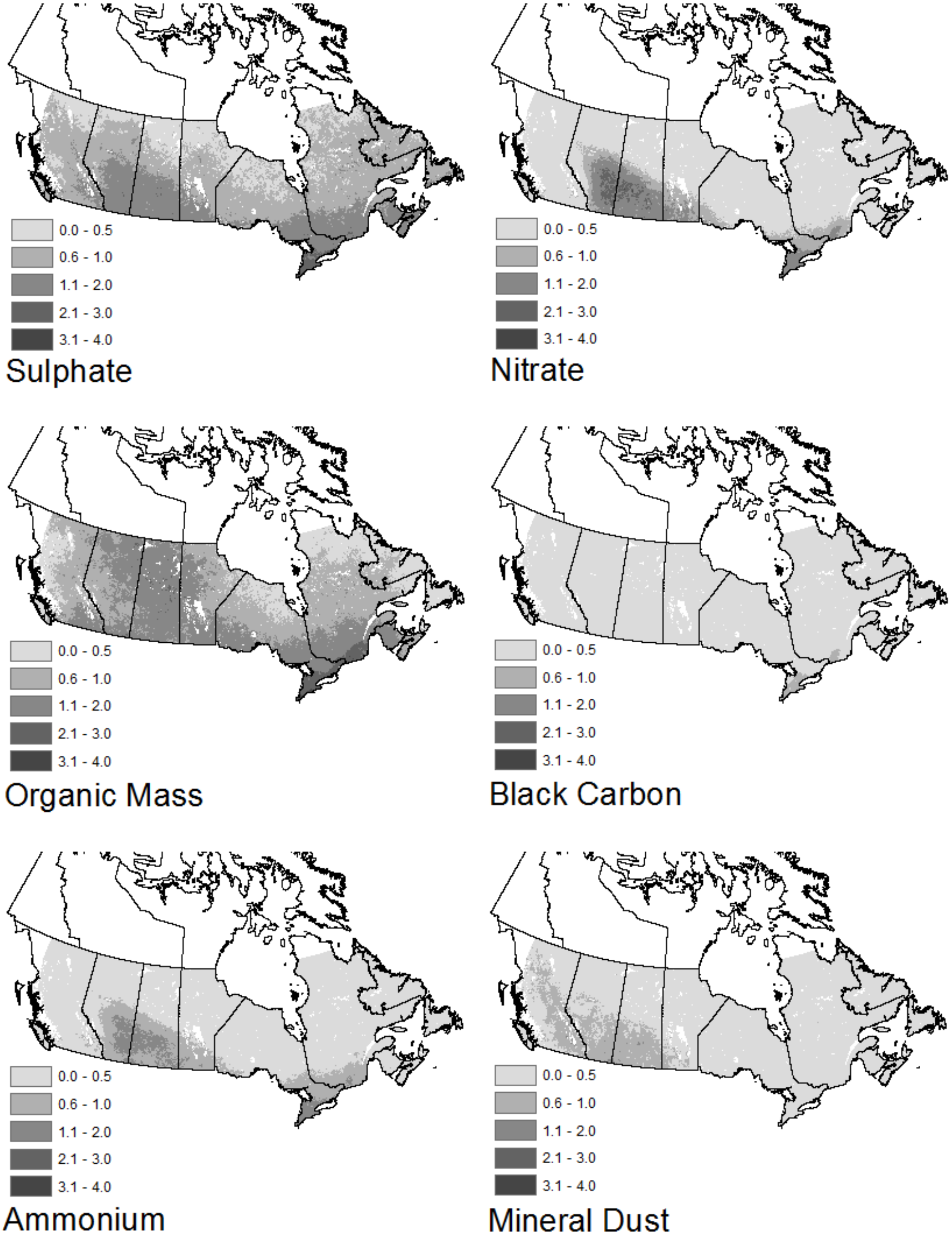

Supplement: Supplementary Information [file srep18916-s1.pdf]
